# Tissue factor-dependent coagulation activation by heme: A thromboelastometry study

**DOI:** 10.1371/journal.pone.0176505

**Published:** 2017-04-24

**Authors:** Gleice Regina de Souza, Bidossessi Wilfried Hounkpe, Maiara Marx Luz Fiusa, Marina Pereira Colella, Joyce M. Annichino-Bizzacchi, Fabiola Traina, Fernando Ferreira Costa, Erich Vinicius De Paula

**Affiliations:** 1 Faculty of Medical Sciences, University of Campinas, Campinas, São Paulo, Brazil; 2 Hematology and Hemotherapy Center, University of Campinas, Campinas, São Paulo, Brazil; 3 University of Sao Paulo, Ribeirao Preto, São Paulo, Brazil; Université Claude Bernard Lyon 1, FRANCE

## Abstract

Heme has been characterized as potent trigger of inflammation. In hemostasis, although heme has been shown to both induce and inhibit different compartments of hemostasis, its net effect on the hemostatic balance, and the biological relevance of these effects remain to be determined. Herein we evaluated the effect of heme on hemostasis using a global assay able to generate clinically relevant data in several other complex hemostatic diseases. Citrated whole blood samples from healthy participants were stimulated by heme or vehicle and incubated for 4h at 37°C. Rotational thromboelastometry was immediately performed. The participation of tissue factor in coagulation activation was evaluated using inhibitory antibody. Heme was able of inducing *ex vivo* coagulation activation in whole blood, affecting predominantly parameters associated with the initial phases of clot formation. This activation effect was at least partially dependent on hematopoietic tissue factor, since the effects of heme were partially abrogated by the inhibition of human tissue factor. In conclusion, using a global hemostasis assay, our study confirmed that heme is able to activate coagulation in whole blood, in a tissue factor-dependent way. These findings could explain the disturbance in hemostatic balance observed in conditions associated with the release of heme such as sickle cell disease.

## Introduction

Clinical and experimental data indicate that hemolytic anemias are associated with a hypercoagulable state [1–4], and that coagulation activation could be a critical element in the pathogenesis of sickle cell disease (SCD) [5,6]. Recently, the release of heme from its intracellular compartment emerged as one of the potential candidates for mediating coagulation activation in these conditions. In addition to its direct toxic effects on cell membranes, heme can act as a danger associated molecular pattern through the activation of TLR4 receptors [7,8], thereby inducing vasocclusion in animal models of SCD [9]. Heme is also able of potently stimulating tissue factor expression in endothelial cells [10] and monocytes [11]. In SCD patients, high levels of free heme have been associated with the risk of acute chest syndrome and vasocclusion [12]. On the other hand, heme has also been shown to have anticoagulant effects in patients with porphyria [13], and to inhibit the activity of specific coagulation factors in *in vitro* studies [14,15]. Accordingly, it was recently hypothesized that heme could trigger coagulation initiation, while also limiting its propagation [16]. A potential explanation for these discrepancies is the well-known limitation of the study of discrete compartments of hemostasis in providing a full appreciation of the hemostatic balance.

Thromboelastometry is a global hemostasis assay that evaluates the interplay between different compartments of hemostasis, as well as the overall kinetics and dynamic of thrombin generation. As such, it could provide novel information about the net effect of heme on whole blood coagulation activation, thus contributing to the understanding of the effects of heme on hemostasis. The biological relevance of this assay has been validated in several clinical scenarios, and it has been previously used to characterize hypercoagulability in hemolytic diseases [2,17]. Here we investigated the effect of heme on coagulation activation using rotational thromboelastometry (Rotem).

## Methods

### Blood sample collection and processing

The study was performed in accordance with the Declaration of Helsinki and approved by the Ethics Committee of State University of Campinas (CEP Unicamp, CAAE 46853115.4.0000.5404). Each participant provided written informed consent form before sample collection. This study was performed with 20 healthy volunteers, homozygotes AA for hemoglobin genotype, without a history of thromboembolic disease or use of drugs that modulated hemostasis. Heme was diluted to an initial working concentration of 700μM in NaOH 0.1M. The pH of this solution was adjusted to 7.4. This solution was filtrated though a 0.22μm filter and immediately used to stimulate whole blood samples, as detailed below. Ultra-filtered water was used for all dilutions. Blood samples were drawn in anticoagulant sodium citrate plastic vacuum tubes (BD Vacutainer Coagulation Tube, 2.7 ml, 0.109 M buffered sodium citrate). Whole blood was immediately incubated with the freshly prepared heme to a final concentration of 30μM (Ref H651-9; Frontier Scientific, USA) for 4h at 37°C, based on the time-course of tissue factor procoagulant activity increase in monocytes and endothelial cells [10,18]. After that, samples were gently mixed and immediately used for Rotem test. This final concentration of heme was used because it mimics the levels of free heme observed in sickle cell disease patients [19,20].

### Rotational thromboelastometry (Rotem) procedures

Rotem was performed using a ROTEM^®^instrument (InnovationsGmbH, Munich, Germany), which evaluates the viscoelastic properties of blood and generate parameters associated with coagulation initiation, kinetics, stability and susceptibility to fibrinolysis. Alll ssays were initiated using a non-activated thromboelastometry (Natem) procedure. Natem is a Rotem-based assay used to assess whole blood clot formation in which no specific activator is used, and only calcium is added to plasma [21,22]. Standard parameters of different stages of clotting were determined and included in the statistical analysis. Clotting time (CT) in seconds (sec) is the time from test start until curve generated by the instrument reaches an amplitude of 2 mm. It is a measure of the initiation phase of coagulation, and is mainly affected by the enzymatic activity of coagulation factors, the concentration of anticoagulants, and by tissue factor expression on circulating cells [22]. Clot formation time (CFT) in sec is defined as the time necessary for the Rotem curve amplitude to increase from 2 to 20 mm. It is mainly dependent on thrombin generation, platelet count and function, and fibrin polymerization [22], and measures reactions that are part of the amplification and propagation phases of coagulation. Maximum clot firmness (MCF) corresponds to the maximum amplitude of the Rotem curve and reflects the mechanically strength of the clot. It depends mainly on platelet count and function, fibrin polymerization and factor XIII activity. In addition to these standard parameters, we also analyzed the effect of heme on parameters derived from the first derivative of the Rotem curve, which corresponds to the velocity signal of the coagulation process. These parameters have been shown to improve the resolution of smaller differences in samples activated by minimal concentrations of tissue factor [23]. These additional parameters are routinely reported by the Rotem software as “research parameters”, and have been extensively used in studies using procoagulant agents such as recombinant factor VIIa, recombinant factor XIII, as well as in other contexts [24–29]. The time to maximum velocity (MAXV-t) is the time from test start until the maximal amplitude of the velocity signal is achieved, and reflects the velocity to maximal clot firmness measured by the method. The area under the velocity curve (AUC) represents the area under this 1^st^ derivative curve (velocity signal) until the point when the maximal amplitude of the Rotem curve (maximum clot firmness) is reached, and indirectly reflects the thrombin generation potential [23,28,30]. All assays were peformed using paired citrated whole blood previously incubated with heme (30μM, pH = 7.4) or vehicle (NaOH 0.1M, pH = 7.4), as described above. Briefly, 300μL of whole blood samples were mixed with 20μL of CaCl_2_ (0.2M, pH = 7.4) in a pre-heated cup at 37°C, and the reaction was started in no more than 17 seconds. Neutralizing anti-tissue factor antibody (Ref 4509, Sekisui Diagnostics, USA) was used at final concentration of 10μg/ml to assess the participation of tissue factor in heme-induced coagulation activation. Non-immune IgG1 isotype (Control IgG mouse, SC-2025, Santa Cruz Biotechnology) was used as negative control at the same concentration.

### Statistical analysis

Statistical analysis was performed using GraphPadPrism 6 (GraphPadPrism Software Inc. San Diego, Califórnia, USA). Wilcoxon two-side test was used to compare quantitative parameters obtained from the same samples exposed to vehicle or heme, with or without inhibition. Results are presented as mean (±SEM). The p-value of < 0.05 was considered significant.

## Results

### Heme induces *ex vivo* coagulation activation in whole blood samples

**Fig 1:**
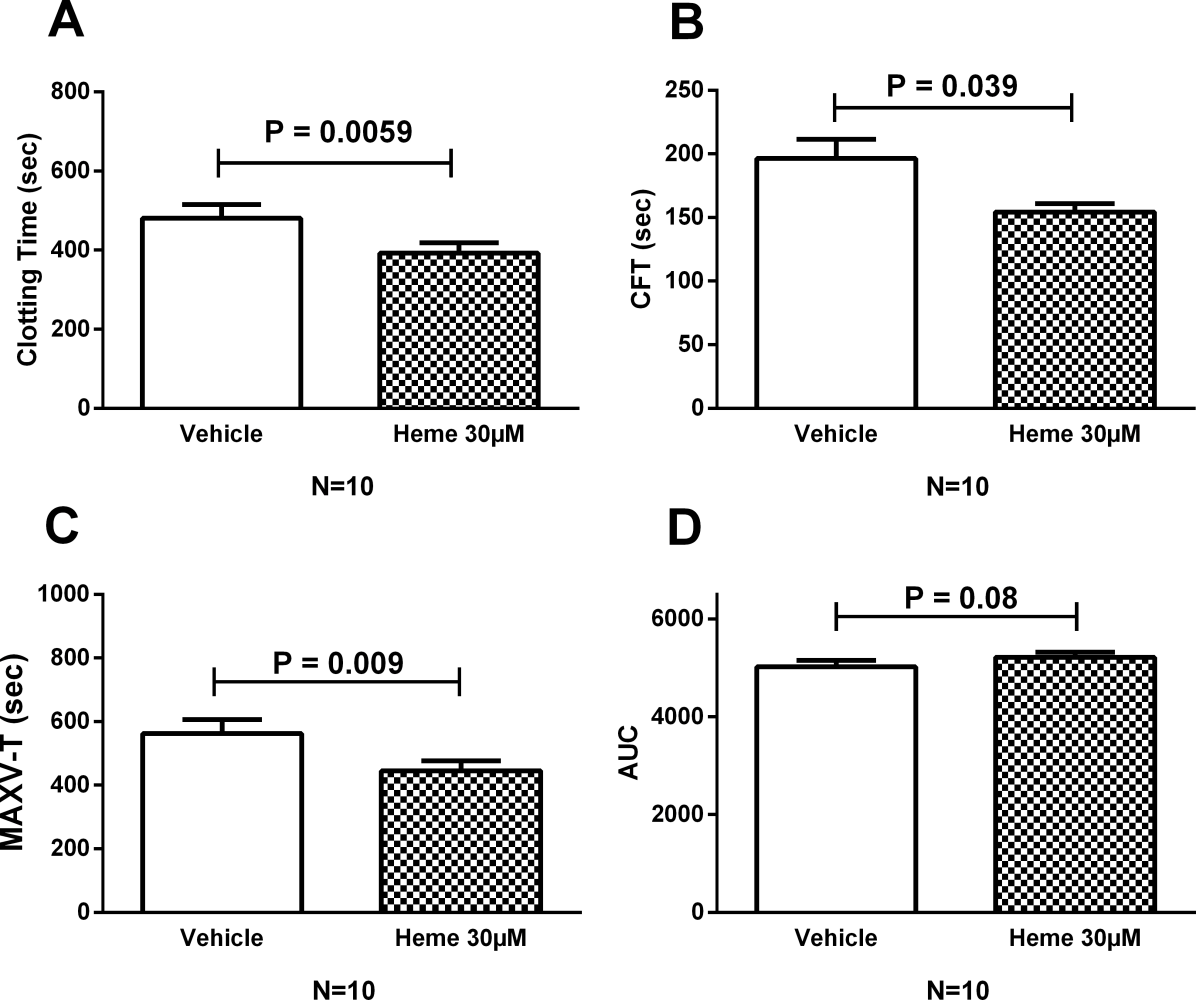
Activation of coagulation and clot formation induced by heme. Comparisons were made for heme *vs* vehicle after 4h of incubation. Histograms were obtained for selected Rotem parameters and show mean (±SEM). Heme induced a significant reduction of clotting time (A), clot formation time (B) and time for maximal velocity (C). No significant change was observed for the area under the curve (D). Two tailed Wilcoxon test was performed and p-values < 0.05 were considered significant. CFT: clot formation time; MAXV-T: time of maximal velocity; AUC: area under curve.

In non-activated thromboelastometry (Natem), heme (30μM) was able of inducing coagulation activation in whole blood samples from healthy participants. After 4h of incubation, heme significantly reduced the CT (Fig 1A) and CFT (Fig 1B) when compared to vehicle. Similar results were obtained with a different preparation of heme (Sigma, Ref 5280). Heme also reduced the MAXV-t (Fig 1C). While a trend towards a higher AUC could be observed in heme-incubated samples (Fig 1D), no difference was observed in maximal clot firmness, MCF (P = 0.054).

### Coagulation activation of whole blood induced by heme is dependent on tissue factor

**Fig 2:**
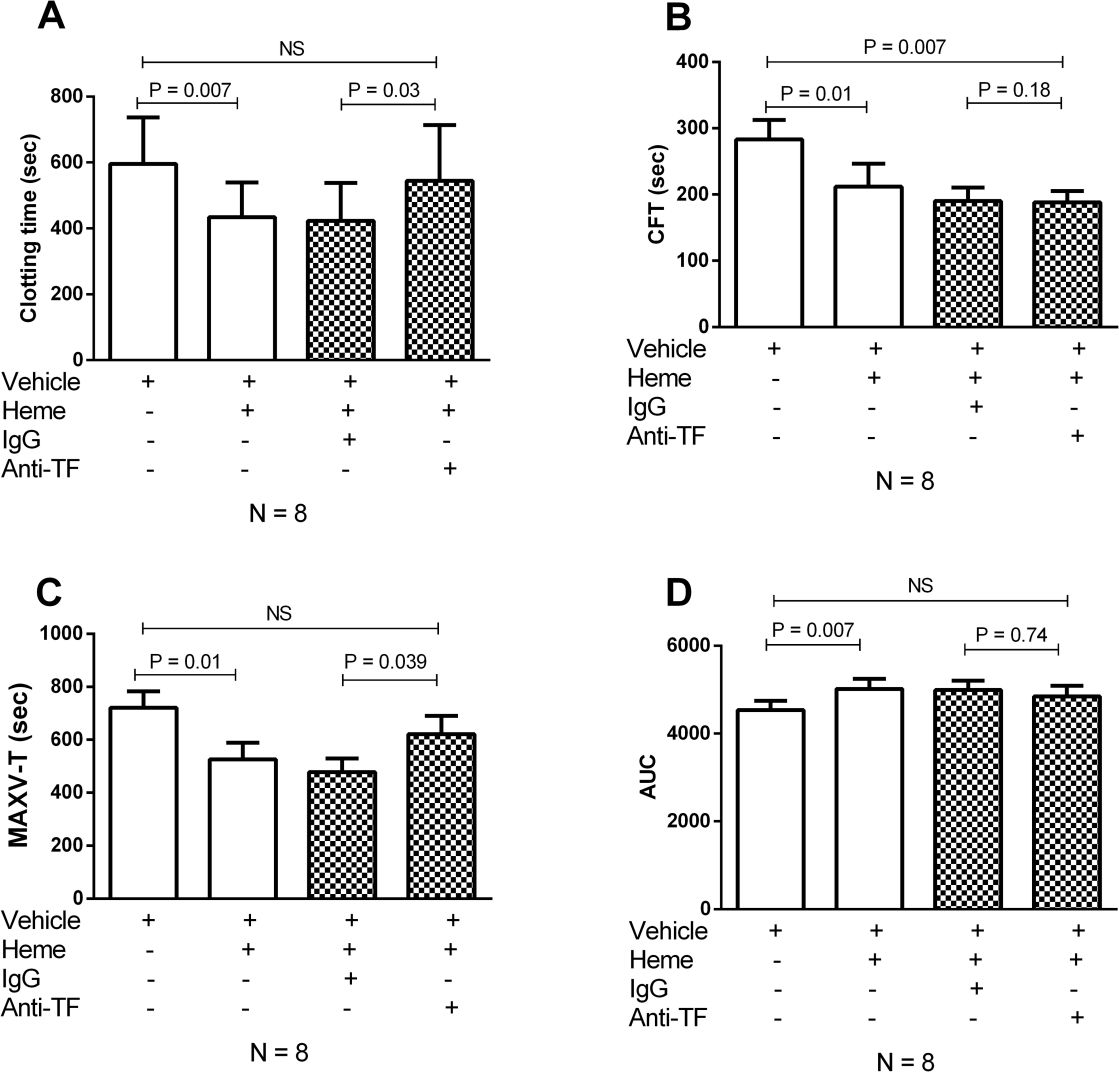
Heme triggered-coagulation activation is tissue factor-dependent. A human inhibitory anti-tissue factor antibody (Ref 4509 Sekisui) significantly delayed the coagulation initiation, prolonging the clotting time (A) and the time to maximal velocity of clot formation (C). No change was observed in the clot formation time (B) or area under curve (D). Two tailed Wilcoxon test was performed and p-values < 0.05 were considered significant. CFT: clot formation time; MAXV-T: time of maximal velocity; AUC: area under curve; Anti-TF: Tissue factor inhibiting antibody.

Since heme has been previously shown to induce tissue factor expression in endothelial cells [31] and monocytes [32], we explored whether the observed alterations induced by heme were dependent on tissue factor expression in our system, by using an inhibitory antibody against tissue factor. When compared to an isotype control, the use of an anti-tissue factor inhibitory antibody abrogated the effect of heme in coagulation activation, resulting in increase in CT (Fig 2A) and in a decrease in MAXV-t (Fig 2C), which represent the two most significant effects of heme compared to vehicle. Inhibition had no effect on CFT (Fig 2B) or in the AUC (Fig 2D). Of note, the effects of heme compared to vehicle were similar to those obtained in the experiment presented in Fig 1, except that in these experiments the trend towards a higher AUC in heme-incubated samples reached statistical significance (Fig 2D).

## Discussion

Increased thrombotic risk is a hallmark of several conditions associated with the extracellular release of heme such as SCD [32] paroxysmal nocturnal hemoglobinuria [33,34], sepsis [35] and intracerebral hemorrhage [36]. Although the mechanisms that underlie the disruption of hemostatic balance in these conditions are not known, the observation that heme can trigger the expression of the physiologic initiator of hemostasis in both endothelial cells [10] and monocytes [11] raises the possibility that it could be involved in the pathogenesis of hypercoagulability in these conditions. Accordingly, the main finding of our study is that heme was able to induce coagulation activation in whole blood, by pathways that are at least partially dependent on tissue factor.

Activation of hemostasis is currently considered a part of innate immunity, participating in the host response to both pathogens, and to sterile danger signals [37]. Heme is an ubiquitous molecule in organisms from all kingdoms of life and in most cells which, over the last decade, has been shown to activate several pathways of innate immunity [7,38–40]. Thus, it is fair to speculate that heme could also represent a mediator of coagulation activation in conditions associated with increased extracellular heme levels [8]. Accordingly, heme has been shown to induce the expression of tissue factor, the physiologic initiator of this process, in both endothelial cells and monocytes [10,11]. Moreover, the biological relevance of this observation was suggested in by the demonstration of tissue factor-dependent coagulation activation in mice treated with heme [32]. In contrast, intravenous hematin (which is very similar to heme preparations used in the former experiments) used in patients with porphyria is not associated with a systemic hypercoagulable state, and has been previously associated with prolongation of coagulation times and to reduced levels of coagulation factors V and VIII [41,42]. Moreover, heme presents complex effects on different compartments of hemostasis that include platelet activation [43] and interactions with VIII [15] and fibrinogen [44] of yet unknown physiological significance. Together, these results illustrate the challenges of addressing the effects of heme on hemostasis, and highlight the importance of methods able to explore its net global effect on this process.

Thromboelastometry is a global hemostasis assay capable to demonstrate signs of hypo- and hypercoagulability in patients with complex alterations of hemostasis such as sepsis, trauma and SCD [45,46]. The use of whole blood samples allows the evaluation of hemostasis in the presence of critical elements to the hemostatic balance such as red blood cells, platelets and leukocytes, in addition to plasma proteins and platelets [17]. Of note, a recent study in SCD demonstrated that relevant parameters of coagulation activation could only be demonstrated in whole blood, but not in plasma [47]. Using this method we demonstrated that heme was able to induce coagulation activation in whole blood samples, as evidenced by shortenings of the CT, CFT and MAXV-t. The first two parameters reflect the initiation and amplification phases of coagulation respectively, while the latter reflects the velocity to reaching maximum clot firmness as measured by the Rotem method (refs Sorensen 2003 e Gorlinger 2016). No significant change was observed in the MCF, which reflects clot strength, and has been shown to be the only Rotem parameter to correlate with the endogenous thrombin potential [48]. These results suggest that the tissue factor-dependent effects of heme could be more relevant to the initial phases of clot formation, with a lower impact on the overall thrombin potential and on clot stability. Interestingly, a similar pattern was observed when the thrombin generation assay was used in a cohort of 92 sickle cell disease patients [49]. Accordingly, we speculate that the fact that TF inhibition only reversed the effects of heme on CT and MAXV-t possibly reflect the higher dependence of these parameters on the initial phases of clot formation when compared to CFT and AUC, which are more dependent on fibrin polymerization and clot stabilization.

In mice, heme-induced coagulation activation was dependent on tissue factor of both endothelial and hematopoietic tissue factor [32]. Using an inhibitory antibody that has been widely used in studies addressing the role of tissue factor, we demonstrated that in human whole blood samples, heme-induced coagulation activation is at least partially dependent on the expression and/or activation of tissue factor. Since in our experimental conditions coagulation activation is dependent solely on circulating blood cells, our results confirm that hematopoietic tissue factor is involved in this process. Accordingly, hematopoietic tissue factor is believed to be the most relevant source for coagulation activation in pathological conditions such as sepsis [50] and SCD [51].

Our system does not intend to model the *in vivo* putative effects of heme in coagulation activation, but only to address its isolated effect on a clinically relevant global hemostasis assay, and to confirm the participation of hematopoietic tissue factor in this process. The effect of free heme on the hemostatic balance *in vivo* is a much more complex scientific question, influenced by variables such as the wide availability of heme scavengers such as hemopexin and albumin in plasma [52], the effects of heme on other discrete compartments of hemostasis [16], and an extremely complex network of cellular pathways that participate in the pathogenesis of hemolytic anemias such as sickle cell disease [53]. Moreover, our results do not exclude the participation of other pathways such as leukocytes, platelets and factor XII in heme-induced coagulation activation *in vivo*.

In conclusion, using a global hemostasis assay capable to detect clinically relevant changes in hemostasis we demonstrated that heme is able to induce coagulation activation of whole blood in a tissue factor-dependent way. These results support the concept that free extracellular heme could play a relevant role in coagulation activation in SCD patients, and in other pathological conditions. Further studies are necessary to elucidate the mechanisms by which heme induces the activation of tissue factor, as well as the *in vivo* relevance of these findings.
